# Tyrosine 129 of the Murine Gammaherpesvirus M2 Protein Is Critical for M2 Function *In Vivo*

**DOI:** 10.1371/journal.pone.0105197

**Published:** 2014-08-14

**Authors:** Udaya S. Rangaswamy, Brigid M. O’Flaherty, Samuel H. Speck

**Affiliations:** 1 Microbiology and Molecular Genetics Graduate Program, Emory University School of Medicine, Atlanta, Georgia, United States of America; 2 Department of Microbiology and Immunology, Emory University School of Medicine, Atlanta, Georgia, United States of America; 3 Emory Vaccine Center, Emory University School of Medicine, Atlanta, Georgia, United States of America; University of Liverpool, United Kingdom

## Abstract

A common strategy shared by all known gammaherpesviruses is their ability to establish a latent infection in lymphocytes – predominantly in B cells. In immunocompromised patients, such as transplant recipients or AIDS patients, gammaherpesvirus infections can lead to the development of lymphoproliferative disease and lymphoid malignancies. The human gamma-herpesviruses, EBV and KSHV, encode proteins that are capable of modulating the host immune signaling machinery, thereby subverting host immune responses. Murine gamma-herpesvirus 68 (MHV68) infection of laboratory strains of mice has proven to be useful small-animal model that shares important pathogenic strategies with the human gamma-herpesviruses. The MHV68 M2 protein is known to manipulate B cell signaling and, dependent on route and dose of virus inoculation, plays a role in both the establishment of latency and virus reactivation. M2 contains two tyrosines that are targets for phosphorylation, and have been shown to interact with the B cell signaling machinery. Here we describe *in vitro* and *in vivo* studies of M2 mutants which reveals that while both tyrosines Y120 and Y129 are required for M2 induction of IL-10 expression from primary murine B cells *in vitro*, only Y129 is critical for reactivation from latency and plasma cell differentiation *in vivo*.

## Introduction

Gammaherpesviruses, members of the *Herpesviridae* family, are lymphotropic viruses that are characterized by their ability to establish latency in lymphocytes - particularly in B cells. The human viruses of this family, Epstein-Barr Virus (EBV) and Kaposi’s Sarcoma associated Herpesvirus (KSHV) are associated with a range of lymphoproliferative diseases and lymphomas in immunocompromised situations (reviewed in [1]). EBV, a member of the lymphocryptovirus genus, is found in all cases of endemic Burkitt’s lyphoma, and is associated with other lymphoid cancers such as Hodgkin’s lymphoma and post-transplant lymphomas, as well as carcinomas such as gastric carcinoma and nasopharyngeal carcinoma (reviewed in [2]). KSHV, a member of the more common rhadinovirus genus, is the etiologic agent of AIDS-related KS, and is also associated with the development of primary effusion lymphoma (PEL) and multicentric Castleman’s disease (reviewed in [3]). However, the strict species tropism of EBV and KSHV greatly hampers detailed studies of viral pathogenesis and host defense *in vivo.* Much of the *in vivo* studies have been accumulated from limited utilization of either small-animal models or primate models.

Murine gammaherpesvirus 68 (MHV68) infection of inbred strains of mice provides a powerful and well-characterized rodent model for analysis of gammaherpesvirus pathogenesis. Infection of mice with MHV68 *via* intranasal inoculation results in a productive acute replication phase in the lung, and subsequently in the spleen – the latter being cleared by 2–3 weeks post-infection (reviewed in [1]). Latency is established primarily in splenic B cells -particularly in naïve, germinal center B cells and memory B cell subsets, as well as macrophages, dendritic cells and lung epithelial cells, as is the case for EBV [4]–[6]. Long-term latency is established predominantly in memory B cells [7]. Recently we have shown that, similar to EBV and KSHV, plasma cells represent the major reactivation reservoir for MHV68 as well [8], strongly linking the conserved strategies utilized by this virus family. Moreover, it was shown that a MHV68 gene called M2 plays a pivotal role in driving differentiation of infected B cells to plasma cells [8].

Sequence analysis and characterization of the MHV68 genome initially identified M2 as a latency associated gene product that bears no homology to any known cellular or viral protein [9], [10]. M2 is crucial for both establishment and reactivation from latency, in a route- and dose-specific manner, but dispensable for acute viral replication in lungs of mice [11], [12]. M2 contains several PxxP motifs that are potential SH3 domain docking sites, as well as two closely-spaced tyrosine residues (Y120 and Y129). Notably, we have previously shown the functional importance of some of these motifs *in vivo* [13]. Although MHV68 M2 does not bear sequence homology to any known gene product, the organization of the functional domains in M2 resembles domains present in the cytoplasmic N- terminal domain of EBV LMP2A. Furthermore, M2 shares with EBV LMP-1 the ability to induce cellular IL-10 expression [14]. We have recently shown that M2 activates the NFAT pathway, similar to K1 of KSHV which has been shown to mimic constitutive ITAM-mediated BCR signaling to activate the NFAT pathway [15], [16]. M2 induces IL10 expression in primary murine B cells [17] and we have shown that this induction occurs via a NFAT-dependent pathway involving IRF4 induction [16]. Depending on the cell type, IL10 activates the JAK-STAT pathway *via* activation of JAK1 (associated with the IL10 receptor alpha chain) and/or TYK2 (associated with the IL10 receptor β chain) and induces the activation of STAT1, STAT3, and, in some cases, STAT5 [11], [18]–[21].

*In vitro* studies have shown that Y120 residue and a C-terminal PxxP motif of M2 are involved in the formation of a complex comprising of M2, Vav1 and Fyn [22]. Additionally, it was shown that Y120 of M2 is constitutively phosphorylated in a B cell line and a mutant virus with Y120 and Y129 mutated to phenylalanine exhibits M2 null phenotype in latency establishment [23]. It was also recently shown that M2 interacts with several cellular proteins via Y120 and/or Y129. While Y120 was predominantly associated with Vav1, p85α subunit of PI3K and NCK1, Y129 was found to interact with PLCγ2, p85α subunit of PI3K and SHP2 [24]. However, the requirement of these individual tyrosine residues in some essential functions of M2, namely establishment of latency, reactivation from latency coupled with plasma cell differentiation, and IL-10 production is unknown. In an earlier study, we have shown that both of these tyrosines are required independently for activation of the NFAT pathway and IRF4 induction [16]. In this study, we aimed to further characterize the roles of Y120 and Y129 in key functional aspects of M2, both *in vitro* and *in vivo*.

## Results

### Tyrosines 120 and 129 of M2 are required for IL10 production and expansion of primary murine B cells in tissue culture

*In vitro* analyses of M2 functions have shown that Y120 of M2 is the predominant residue required for formation of a trimolecular complex of M2 with the Src kinases Fyn and Vav. It was also shown that Y120 is constitutively phosphorylated upon expression in a B cell line as well as a non-hematopoietic cell line [22]. However, in our recent studies on the signaling of M2, both Y120 and Y129 were singly required for activation of the NFAT pathway by M2. Both Y120 and Y129 were also required for induction of the plasma cell-associated transcription factor, IRF4 [16]. Since M2 induction of IRF4 leads to IL-10 induction in B cells, we wanted to further study the roles of the individual tyrosines of M2 in IL-10 production in a more physiological context, namely primary murine B cells. We generated retroviral constructs in which each of the tyrosines at positions 120 and 129 of M2 was mutated to a phenylalanine. B cells were isolated from the spleens of naïve C57Bl/6 mice and transduced with the recombinant MSCV retroviruses expressing either the M2/Y120F or the M2/Y129F mutant [recombinant MSCV expressing either wild type M2 or a null mutant of M2 (M2stop; in which a translation stop codon was introduced in place of residue 13 in the M2 open reading frame) were used as positive and negative controls, respectively]. The expression of an IRES-Thy1.1 cassette downstream of the M2 open reading frame served as a surrogate marker to monitor retroviral transduction and M2 expression. To assure that the Y120F and Y129F mutant M2 proteins were expressed from the pMSCV-IRES-Thy1.1 (pMIT) vector, we assessed M2 expression by immunoblotting following transfection of the WT and M2 mutant retroviral vectors into 293T cells. Notably, both the Y120F and Y129F M2 mutants were expressed to similar levels as wild type M2 (Fig. S1).

**Figure 1:**
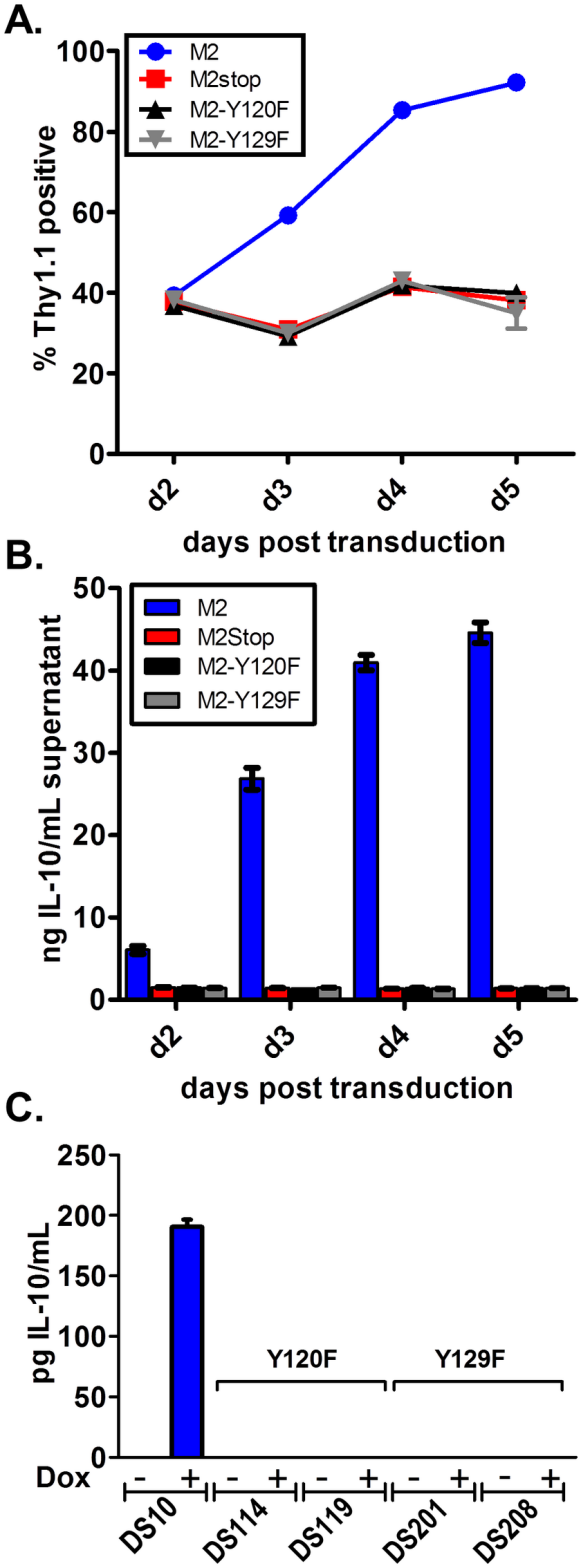
Y120 and Y129 of M2 are required for expansion of primary murine B cells and for IL-10 production. (A and B) Primary murine B cells were transduced with retroviral vectors encoding Y120F or Y129F mutations of M2, wild type M2 or M2stop as a negative control. On days 2–5 post transduction, the cells were analyzed by (A) flow cytometry to monitor Thy1.1 expression, and (B) the supernatants from cells in (A) were analyzed for IL-10 secretion by ELISA. (C) 1–2×10^6^ cells each of the Y120F or Y129F inducible cell lines described in materials and methods were induced with doxycycline for 48 hours and supernatants were harvested for measurement of IL-10 secretion by ELISA. Data shown is a representative of one experiment with three replicates per condition. Each experiment was performed at least twice with at least three independent replicates per experiment.

Upon retroviral transduction, primary murine B cells expressing wild type M2 expanded in culture over time - consistent with our previous results (Fig. 1A) [17]. However, mutation of either Y120 or Y129 to phenylalanine abolished M2 mediated expansion of Thy1.1 expressing cells, with both mutants expressing levels of Thy1.1 similar to that of the negative control M2stop (Fig. 1A). Furthermore, as we have previously shown [17], supernatants from B cells transduced with the wild-type M2 retrovirus contained high levels of IL-10 compared to M2stop, while mutation of either tyrosine completely abrogated IL-10 production (Fig. 1B). We extended these analyses to assess IL-10 induction in M2 inducible cell lines, generated as previously described [16]. Consistent with the data in primary B cells, there was no IL-10 secretion upon doxycycline induction of inducible cell lines expressing either the M2/Y120F or M2/Y129F mutant (Fig. 1C). These data indicate that although Y120 is the predominant tyrosine required for interactions with Src kinases and Vav, both Y120 and Y129 are required for IL-10 production and expansion of primary B cells.

### M2 induced IL-10 primarily signals through pSTAT3, but not pSTAT1 or pSTAT5

**Figure 2:**
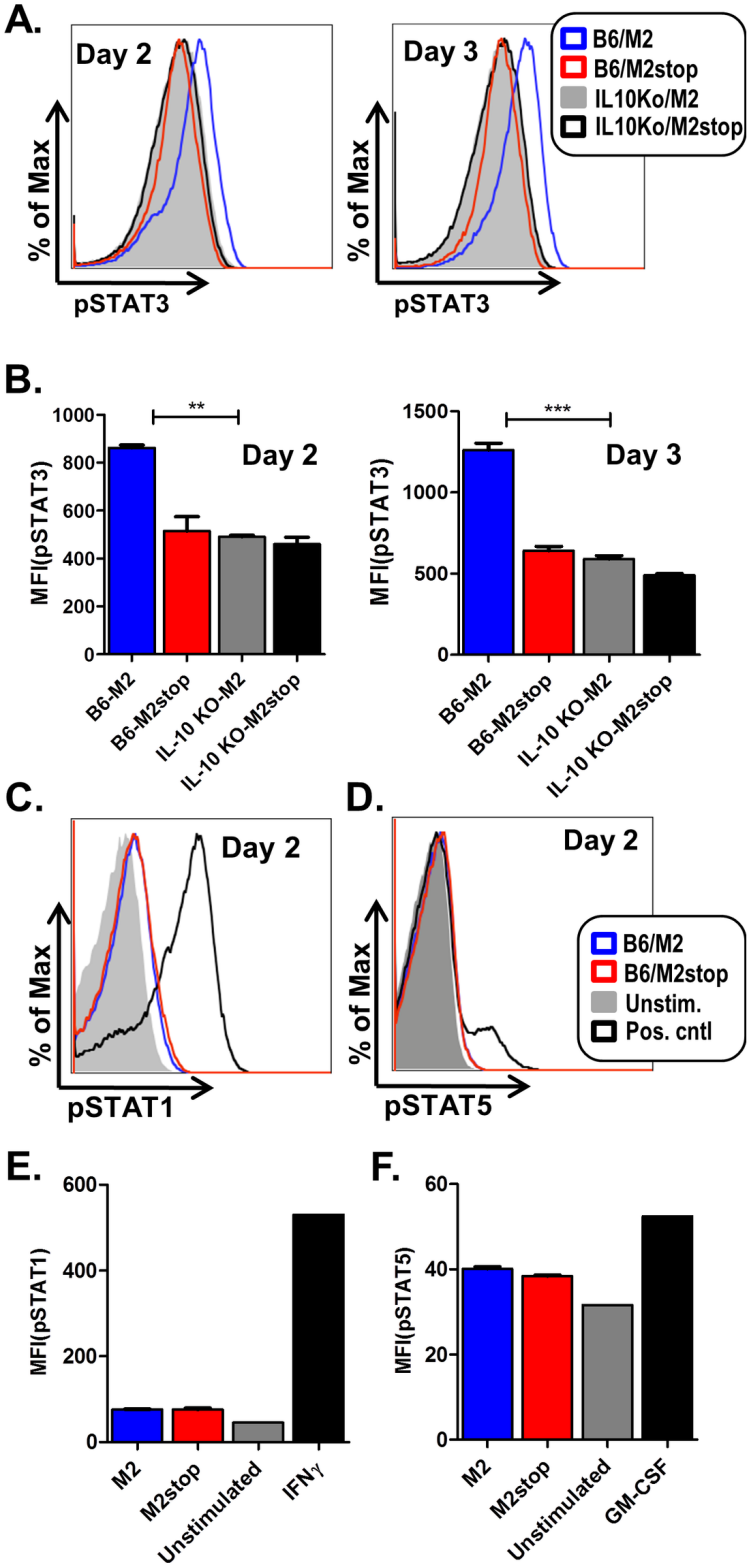
M2 induced IL-10 signals through positive feedback involving STAT3, but not STAT1 or STAT5. (A–F) Primary B cells from either wild type C57BL6 (B6) mice or IL10−/− mice (as indicated) were transduced with retroviral vectors encoding either M2 or M2stop. On days 2 and 3 post transduction, the cells were analyzed by phosphoflow as described in materials and methods. (A) B cells from B6 or IL10−/− mice transduced as mentioned above were analyzed for pSTAT3 levels after gating on live cells by forward and side scatter characteristics. Representative histograms of pSTAT3 levels, one each on day 2 and day 3 are shown. (B) Mean Fluorescent Intensities (MFIs) of pSTAT3 fluorescence from three independent replicates are shown for each condition shown in A. (C–D) B cells from B6 mice transduced as mentioned above were analyzed for pSTAT1 or pSTAT5 levels, respectively. Positive control for pSTAT1 induction is IFNγ treatment of cells at 100 ng/mL for 15 minutes and positive control for pSTAT5 induction is treatment of cells with GM-CSF at 100 ng/mL for 15 minutes. Representative histogram from day 2 post-transduction for each condition is shown. (E–F) Mean Fluorescent Intensities (MFIs) of pSTAT1 or pSTAT5 fluorescence from average of three independent replicates are shown for each condition shown in C and D. The experiments were done at least three times with three replicates in each experiment.

Several studies have shown that IL-10 induces positive feedback signaling by acting through the IL-10R, which signals primarily through activation of STAT3, as well as STAT1 or STAT5 in some cell types (reviewed in [25], [18], [21]). To understand whether M2 induced IL-10 signals using the same pathways, we measured activation of STAT3 by determining the levels of phosphorylated STAT3 by flow cytometry. Primary murine B cells were transduced with retroviruses expressing either M2, M2stop or the tyrosine mutants and the levels of pSTAT3 were measured by phosphoflow. On days 2 and 3 post-transduction, M2 transduced cells had higher levels of pSTAT3 compared to M2stop or the tyrosine mutant transduced cells (Figure 2A and 2B). As expected, negative control B cells from IL-10 knockout mice that were transduced with either M2 or M2stop expressing retroviruses failed to up-regulate pSTAT3 (Figure 2A and 2B). Since IL-10 can signal via pSTAT1 and pSTAT5 in some cell types, we also looked at pSTAT1 and pSTAT5 levels upon M2 expression. Notably, M2 transduced B cells failed to up-regulate either pSTAT1 (Figure 2C and 2E) or pSTAT5 (Figure 2D and 2F). In contrast, positive controls in each experiment, namely, IFNγ and GM-CSF induced pSTAT1 (Figure 2C and 2E) and pSTAT5 (Figure 2D and 2F), respectively, had higher levels of pSTAT1 or pSTAT5 compared to unstimulated samples. It is to be noted that pSTAT5 is predominantly activated in T cells in response to IL-2 [26] and in myeloid cells in response to GM-CSF [27]. Therefore, we observed only a modest increase in pSTAT5 levels upon GM-CSF treatment compared to unstimulated B cells. Additionally, pSTAT1 and pSTAT5 levels are moderately higher in both M2 as well as M2stop transduced samples (Figure 2E and 2F) compared to unstimulated samples indicating that LPS stimulation can activate some levels of pSTAT1 and pSTAT5 in murine B cells (note- to efficiently retrovirally transduce primary murine B cells we transiently stimulated these B cell cultures overnight with LPS).

**Figure 3:**
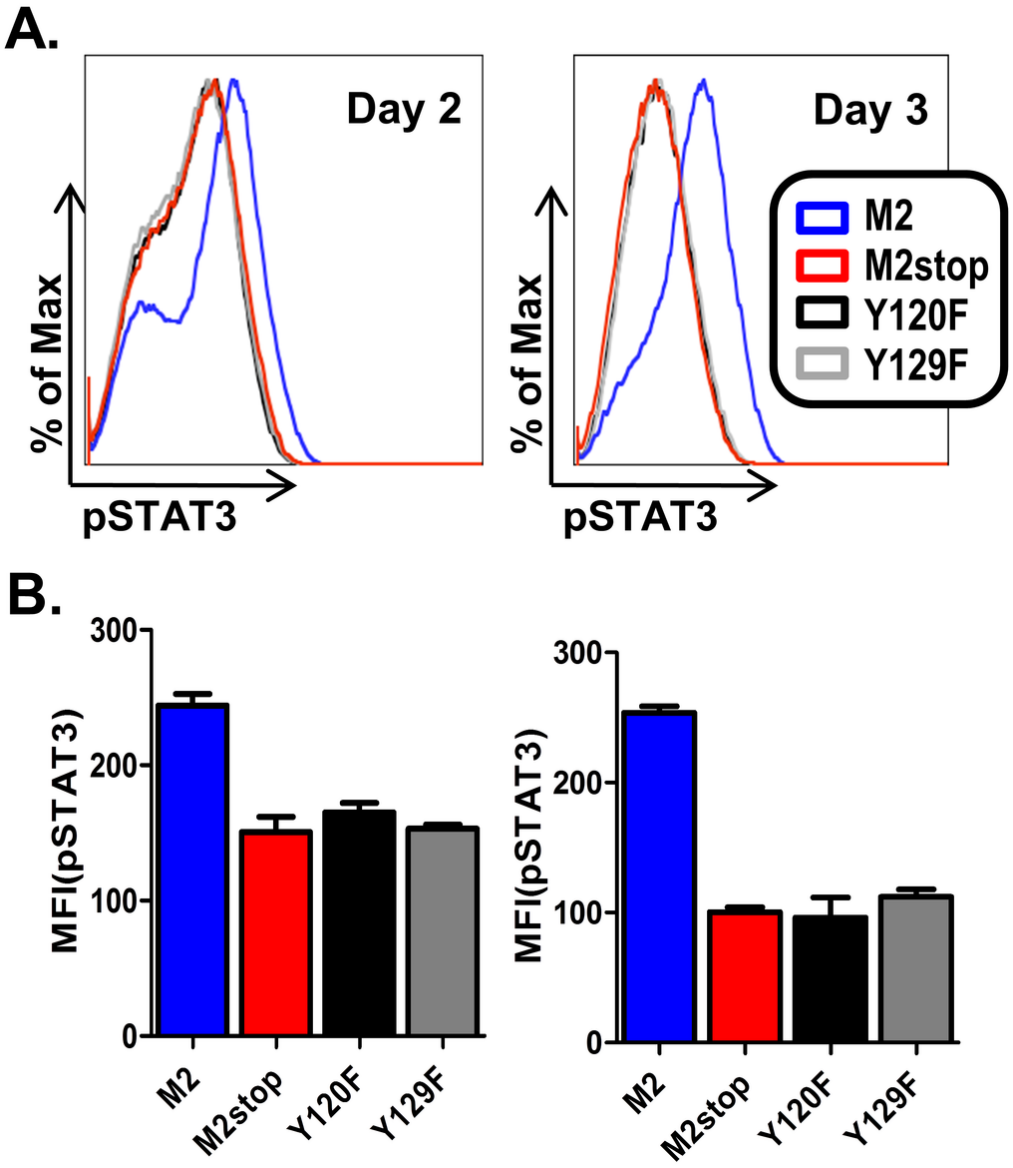
Y120F and Y129F do not induce phosphorylation of STAT3. (A–B) Primary B cells from wild type C57BL6 (B6) mice were transduced with retroviral vectors encoding either Y120F, Y129F, wild type M2 or M2stop. On days 2 and 3 post transduction, the cells were analyzed by phosphoflow as described in materials and methods. (A) B cells from B6 mice transduced as mentioned above were analyzed for pSTAT3 levels after gating on live cells by forward and side scatter characteristics. Representative histograms of pSTAT3 levels, one each on day 2 and day 3 are shown. (B) Mean Fluorescent Intensities (MFIs) of pSTAT3 fluorescence from three independent replicates are shown for each condition shown in A.

Based on the requirement of both Y120 and Y129 for induction of IL-10 expression, we extended these analyses to assess STAT3 phosphorylation with the M2/Y120F and M2/Y129F mutants. As shown in Figure 3A and Figure 3B, both the M2/Y120F and M2/Y129F mutants failed to induce phosphorylation of STAT3. This is consistent with our hypothesis that the pSTAT3 levels we observe upon M2 expression are a result of M2-mediated IL-10 induction feeding back upon the IL-10R to activate pSTAT3, rather than M2 itself activating STAT3 to increase pSTAT3 levels. Furthermore, when we blocked IL-10 signaling with an antibody against the IL-10R, we failed to see an increase in the level of pSTAT3 (data not shown). Together, these results indicate that M2 induced IL-10 signals predominantly through pSTAT3 in primary murine B cells, and that M2 requires both Y120 and Y129 for induction of IL-10 expression in primary murine B cells.

### Y129, but not Y120, of M2 is required for an efficient reactivation from latency *in vivo*

Given the requirement of both Y120 and Y129 in M2-mediated functions *in vitro* in primary murine B cells, we sought to further determine the requirement of each of these tyrosines *in vivo* in the pathogenesis of MHV68– focusing on establishment of latency and reactivation. Notably, *Pires de Miranda et al* have shown that a mutant virus with both tyrosines Y120 and Y129 mutated to phenylalanine exhibits a severe defect in latency similar to that of an M2 null virus, indicating that at least one or both of these tyrosines are required for M2 mediated functions *in vivo* [23]. To identify the requirement of each of the tyrosines, we generated mutant viruses in which either Y120 or Y129 was mutated to phenylalanine (designated Y120F.HY and Y129.HY, respectively). We used the λRed recombineering system utilizing the galK selection method described by *Warming et al* [28] to generate the mutant viruses. To this end, we utilized as the starting platform, the MHV68-H2bYFP-BAC which harbors an EYFP cassette fused to the histone H2B open reading frame, which allows infected B cells to be monitored by flow cytometry and immunofluorescence. This recombinant MHV68 has been extensively characterized and behaves like wild-type MHV68 [29]. The introduction of these mutations into the viral genome was confirmed by PCR of the M2 region derived from BAC-purified DNA, as well as from DNA obtained from latently infected splenocytes to rule the presence of any revertants that had restored wild-type M2 sequence. To further confirm the absence of unwanted mutations or insertions within the region of homologous recombination, we performed Southern blotting analyses combined with RFLP analyses (Figure S2). We have previously described a M2null virus, and a marker rescue virus termed M2stop.HY and M2MR.HY, respectively. These viruses were also created using the MHV68-H2bYFP background and behave similarly to their non-transgenic counterparts [16].

**Figure 4:**
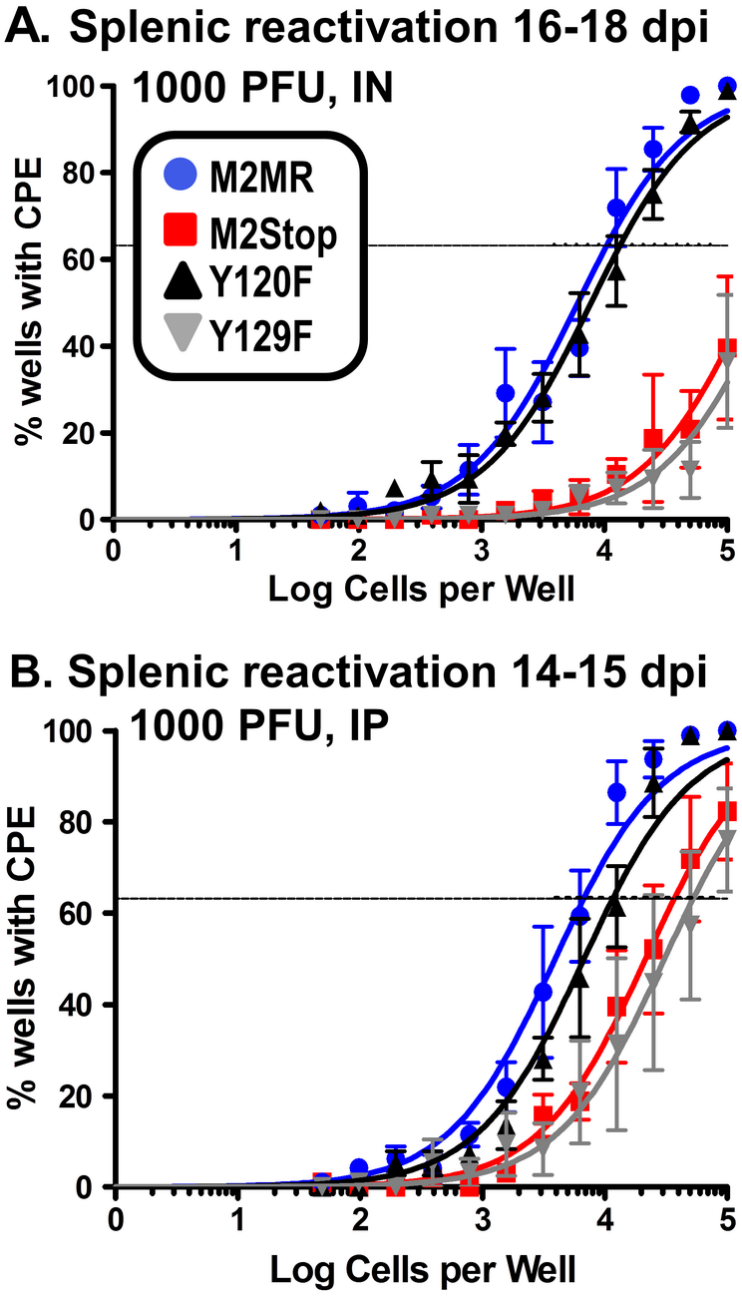
M2 requires Y129, but not Y120, for efficient establishment of latency and reactivation from latency in splenic B cells. (A–D) C57BL6 mice were infected with 1000PFU of the indicated virus either intranasally (A–B) or intraperitoneally (C–D) and splenocytes were harvested at indicated time points. (A and C) The frequency of splenocytes harboring viral genomes were estimated by a limiting dilution, nested PCR assay as described in materials and methods. (B and D) The frequency of splenocytes reactivating virus in a limiting dilution *ex vivo* assay were analyzed on days 14–21 post plating, as described in materials and methods. For IN experiments (A and B), mice were harvested on 16–18 dpi and for the IP experiments (C and D) mice were harvested on 14–15 dpi. For the *ex-vivo* reactivation assay (B and D), intact cells were serially diluted and plated on a MEF monolayer alongside mechanically disrupted cells (as a measure of preformed infectious virus present), as described in materials and methods. The level of preformed infectious virus was below the limit of detection in the above experiments and is therefore not shown in the figures. Results shown in panels A and C are from 3 individual experiments with 3–5 mice per group. Results shown in panels B and D are from 4 individual experiments with 3–5 mice per group.

Previous studies have shown that the requirements for M2 in both the establishment of latency and reactivation from latency are dependent on the dose and route of infection [12]. Low dose intranasal (IN) inoculation with 100 PFU of M2stop virus resulted in a severe latency defect which was overcome by a higher dose of inoculation at 4×10^5^ PFU, or by intraperitoneal (IP) inoculation at a dose of 100 PFU [11], [12]. Because the transgenic H2BYFP viruses have been characterized primarily with a 1000 PFU dose administered either IN or IP, we chose to look at the requirement forY120 and Y129 at 1000 PFU. Since the kinetics of IP infection are faster than that observed following IN infection, we analyzed days 14–15 post-infection compared to days 16–18 post infection for IN infections. We initially examined virus reactivation from latency following intranasal inoculation. Consistent with our previous characterization of M2 null mutants, we observed a severe defect in M2 null virus (M2stop.HY) reactivation compared to marker rescue virus (M2MR.HY) (estimated to be ∼24-fold lower) (Figure 4A). Surprisingly, reactivation of the Y120F mutant was very similar to the marker rescue virus, while reactivation of the Y129F mutant was similar to the M2 null mutant (Figure 4A). Analysis of virus reactivation following intraperitoneal inoculation, as expected, showed a more modest phenotype of the M2 null mutant (Figure 4B). Notably, the Y120F mutant again was very similar to the marker rescue virus (1 in 6729 cells reactivating virus from M2MR.HY infected mice vs 1 in 11614 cells from Y120F.HY infected mice), while the Y129F mutant behaved like the M2 null mutant (a 3–5 fold defect in reactivation compared to M2MR) (Figure 4B). Of note, we did not observe significant levels of preformed infectious virus in any of the assays described (data not shown) – thus indicating that the cytopathic effect observed in the reactivation analyses was due to reactivation of latently infected cells. To assess whether the absence of a strong phenotype with the Y120F mutant could be due to reversion of the Y120F mutation back to the wild type sequence, we PCR amplified and sequenced the M2 gene from latently infected splenocytes. These analyses confirmed detected only the presence of Y120F mutation (data not shown). Taken together, these analyses show that despite an absolute requirement for both Y120 and Y129 in M2-mediated functions *in vitro*, only Y129 appears to play a critical role for M2-mediated functions *in vivo*.

### Y129, but not Y120, of M2 is required for efficient access of infected B cells to the plasma cell reservoir

**Figure 5:**
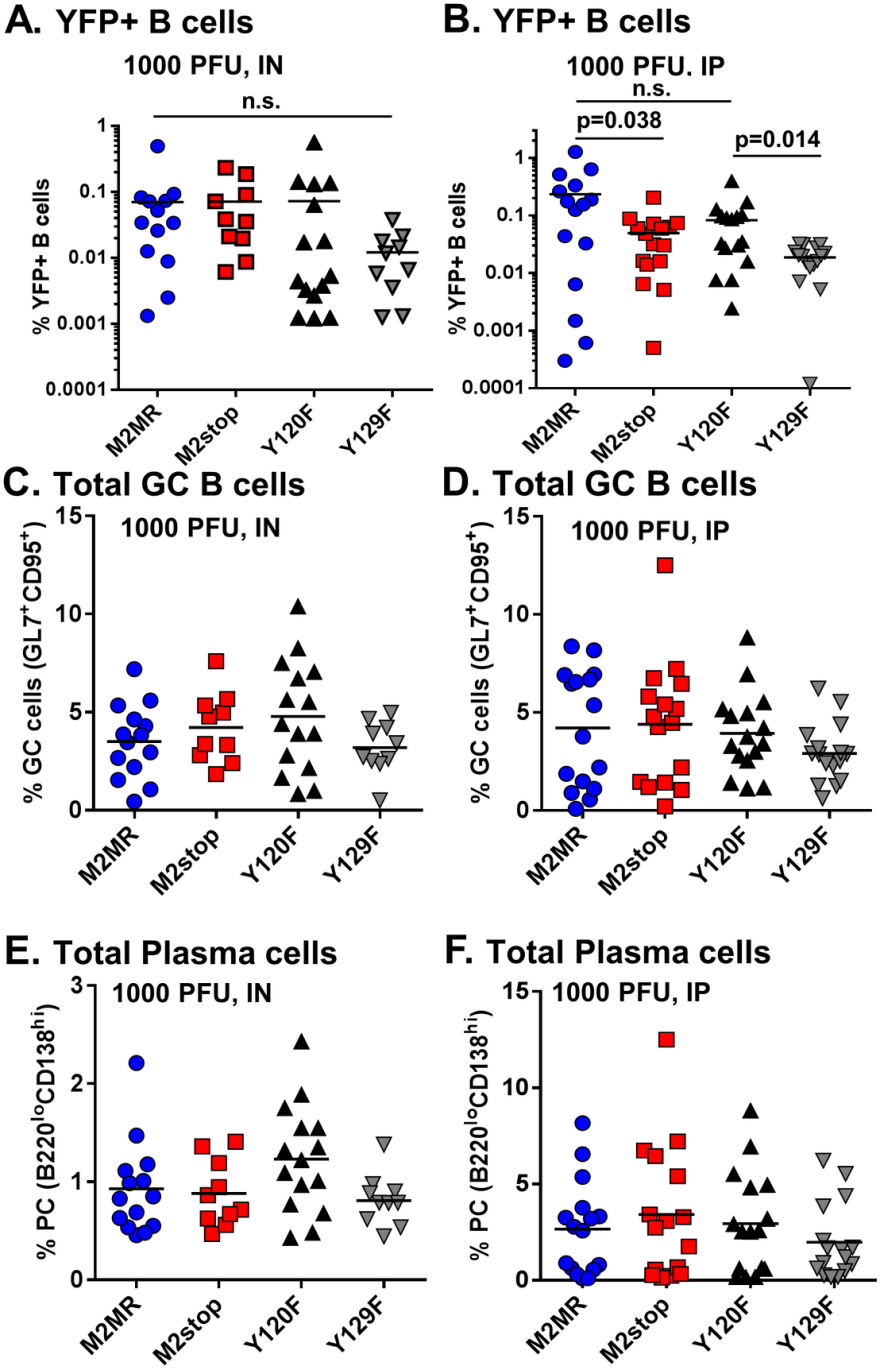
Infections with Y120F or Y129F mutant viruses do not have an effect on the total number of germinal center B cells or plasma cells. (A–F) C57BL6 mice were infected with 1000PFU of the indicated virus either intranasally (A, C and E) or intraperitoneally (B, D and F) and splenocytes were harvested at indicated time points. Splenocytes were stained for flow cytometry as described in materials and methods. (A and B) Mice were harvested at the indicated time points after either IN (A) or IP (B) inoculation. Splenocytes were analyzed for expression of YFP positive B cells after gating out doublet cells, followed by gating on dump^−^ (a mix containing CD3, CD4 and CD8 antibodies to gate out T cells) and B220^+^ cells. Data shown represents the frequency of Dump^−^/B220^+^/YFP^+^ cells. (C–F) Splenocytes were stained as above for determining the frequency of total number of cells with a germinal center phenotype, defined as dump^−/^B220^+^/CD95^+^GL7^+^ cells in panel C and D or, total number of cells with plasma cell phenotype, defined as dump^−^/B220^l^°CD138^hi^ cells in panel E and F. Each data point refers to an individual mouse and the plots depict total mice from 3–4 experiments with 3–5 mice per group per experiment. *P* values were determined using a two-tailed paired Student’s *t* test (n.s., not significant). Note for the data presented in panels C–F that there were no statically significant differences between experimental groups.

Previously, we have shown that M2 is required for plasma cell differentiation of infected cells and that plasma cells are the major reactivation reservoir in infected spleens [8]. Using a transgenic virus that can mark infected cells (M2stop.YFP), it was shown that upon infection with a M2 null virus, there was a significant decrease in the frequency of infected cells with a plasma cell phenotype. Since the viruses used in this study express an H2bYFP cassette, we can identify the frequencies of infected cells by monitoring YFP expression by flow cytometry of splenocytes recovered from infected mice. On days 16–18 post infection via IN route, M2MR.HY, M2stop.HY and Y120F.HY had similar frequencies of YFP+ B cells (doublet-discriminated/CD3^−^CD4^−^CD8^−/^B220^+^), whereas Y129F.HY infected mice had a lower frequency of infected B cells (Figure 5A). It was surprising that the Y129F.HY infection appears to have a significant defect in the frequency of infected cells compared to M2stop.HY. This may indicate that expression of this mutant M2 is more deleterious to establishment of MHV68 infection than the absence of any M2 expression (i.e., the M2 Y129F mutant may function as a dominant negative that interferes with viral latency). Notably, the Y129F.HY mutant did not have any defect in growth - growing to similar titers as that of M2MR.HY and M2stop.HY (data not shown). Importantly, taken together these analyses demonstrate that both the M2stop.HY and Y129F.HY mutants have a pronounced reactivation defect and a mild to moderate impact on establishment of latency. In contrast, mutation of Y120 has little impact on MHV68 latency and reactivation.

While the frequency of M2stop.HY and M2MR.HY infected cells was very similar following IN infection, we did observe a significant decrease in the YFP+ B cell fraction upon infection with the M2stop.HY compared to M2MR.HY following IP inoculation (0.05% in M2stop.HY compared to 0.24% in M2MR.HY) (Figure 5B). We believe that this disparity may be due to the high mouse-to-mouse variability we observe with the YFP marking of infected cells (as observed here in Figures 5A and 5B and also in references [8], [29], [30]) – indeed the frequency of marker rescue infected B cells following IN inoculation was overall lower than we have previously observed with wild type MHV68, while the frequencies of marker rescue infected B cells following IP inoculation were more reflective of the frequency observed with wild type MHV68. Despite variable numbers of YFP+ B cells observed with the different viruses, there was little differences noted in the total number of germinal center (GC) B cells (CD3^−^CD4^−^CD8^−/^B220^+^, GL7^+^CD95^+^) and plasma cells (CD3^−^CD4^−^CD8^−/^B220^l^°CD138^hi^) upon infection via either IN or IP route (Figures 5C–5F). In our earlier analyses of infections with M2stop.YFP virus, we noticed a decreased number of total GC cells upon M2stop.YFP infection at a dose of 100PFU administered via IN route [8]. Thus, the data in Figure 5C indicates that the defect in eliciting a strong germinal center response at 100PFU IN can be overcome by infecting with a 1000PFU.

**Figure 6:**
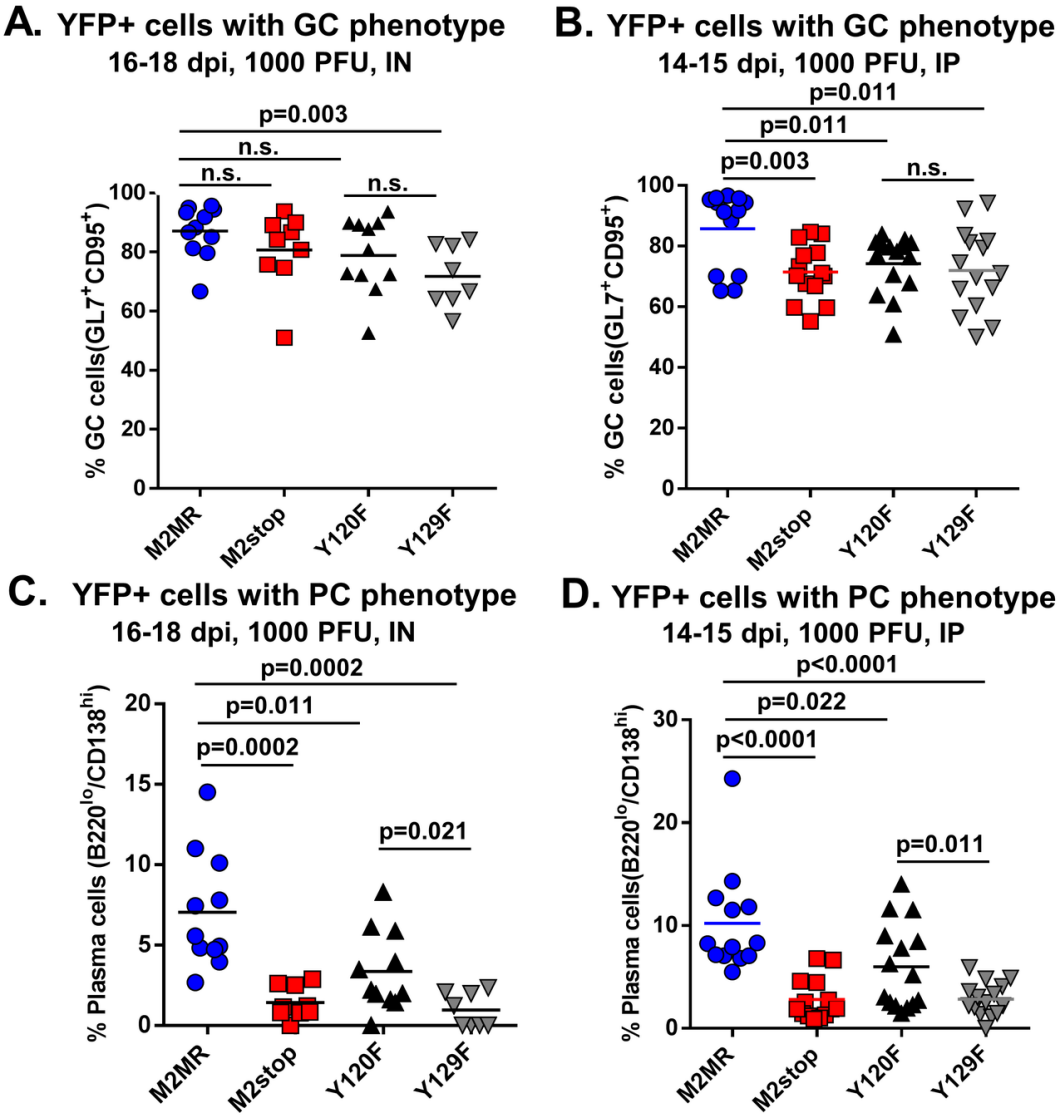
Y129, but not Y120, of M2 is required for differentiation of infected B cells to plasma cells. (A–D) C57BL6 mice were infected with 1000PFU of the indicated virus either intranasally (A and C) or intraperitoneally (B and D) and splenocytes were harvested at indicated time points. Splenocytes were stained for flow cytometry as described in materials and methods. (A and B) Mice were harvested at the indicated time points after either IN (A) or IP (B) inoculation. YFP positive cells with a germinal center phenotype were analyzed as follows- doublet discriminated cells were gated on dump^−^ (a mix containing CD3, CD4 and CD8 antibodies to gate out T cells)/B220^+^/YFP+/CD95^+^GL7^+^. Data shown represents the frequency of Dump^−^/B220^+^/YFP^+^ cells that are CD95^+^GL7^+^, representing the infected cells with a germinal center phenotype. (C and D) Mice were harvested at the indicated time points after either IN (C) or IP (D) inoculation. YFP positive cells with a plasma cell phenotype were analyzed as follows- doublet discriminated cells were gated on dump^−^ (a mix containing CD3, CD4 and CD8 antibodies to gate out T cells)/YFP+/B220^l^°CD138^hi^. Data shown represents the frequency of Dump^−^/YFP^+^ cells that are B220^l^°CD138^hi^ representing the infected cells with a plasma cell phenotype. Each data point refers to an individual mouse and the plots depict total mice from 3–4 experiments with 3–5 mice per group per experiment. *P* values were determined using a two-tailed paired Student’s *t* test (n.s., not significant).

Based on the results from Figure 4, we expected that the Y129F.HY virus infected cells would have a defect in plasma cell differentiation similar to a M2 null virus, but that the frequency of Y120F.HY infected plasma cells would be similar to marker rescue virus levels. To further characterize the requirement of the individual tyrosine residues in their ability to efficiently infect GC cells and plasma cells, we analyzed YFP+ cells exhibiting either a GC or plasma cell phenotype. Upon IN or IP infection of mice with the M2MR.HY, M2stop.HY, Y120F.HY or Y129F.HY viruses, the frequency of YFP+ cells with a GC phenotype was similar between the different groups – with the majority of the virus infected cells exhibiting a GC phenotype in all the cases (Figure 6A and 6B). In contrast, there was a striking difference in the frequency of YFP+ cells with a plasma cell phenotype in M2stop.HY virus infected cells compared to the M2MR.HY infected cells upon either IN or IP inoculation (Figure 6C and 6D) - ∼7% of M2MR.HY infected cells vs 1.4% in M2stop.HY infected cells via IN route and ∼10% of M2MR.HY infected cells vs 2.8% in M2stop.HY infected cells via IP route. In addition, the Y120F.HY mutant exhibited a ∼2-fold decrease in the fraction of infected cells with plasma cell phenotype, indicating that the Y120 residue may be partially required for infected cells to differentiate into plasma cells. Consistent with our above results, the Y129F.HY virus was similar to that of the M2stop.HY virus exhibiting a significant defect in infection of plasma cells (Figure 6C and 6D). Taken together, the data strongly indicates that the majority of M2 mediated functions *in vivo* require Y129.

## Discussion

From prior studies on the functions of M2, it is hypothesized that M2 primarily functions as a molecular scaffold - acting to relay signals that mimic those involved in BCR signaling [16], [22]–[24], [31]–[33]. M2 contains nine PxxP motifs capable of binding cellular proteins with SH3 domains, as well as two tyrosine residues that upon phosphorylation are capable of binding SH2 domain containing proteins. Several candidate interacting partners for M2 have been identified, but the importance of these interactions *in vivo* in the context of MHV68 infection remains unclear. Identifying specific domains in M2 that are involved in these interactions, and the role of these domains in M2 function *in vivo*, is crucial to gaining insights into how MHV68 utilizes M2 to hijack the host cell signaling machinery to its own benefit. As a first step towards this, we sought to identify the requirement of each of these tyrosines in functions mediated by M2.

In this study, we have identified Y129 as the critical tyrosine residue important for the function of the MHV68 gene M2 *in vivo*. Surprisingly, mutation of Y120 to phenylalanine had very little effect on the functions of M2 *in vivo* – only a moderate defect in the differentiation of infected B cells to plasma cells (Figure 6C). This strongly indicates that Y120 is mostly dispensable for M2 mediated functions *in vivo*. However, *in vitro* Y120 was required for M2 induction of the NFAT pathway, IRF4 [16] and IL-10 levels (Figure 1B). Notably, Y120 has been shown to be constitutively phosphorylated in a B cell line and to be the key interacting residue of M2 with Vav1, Fyn and NCK1 [23], [24]. There are several possibilities that may explain the discrepancy between the in vitro and in vivo analyses of the Y120F mutant, which are not mutually exclusive: (i) *in vivo*, in the context of the viral genome, either a host factor and/or a viral factor compensates for the loss of Y120– perhaps by facilitating formation of the appropriate signaling complex; (ii) constitutive phosphorylation of Y120 is required for “tonic” signaling in B cells expressing M2, and that this tonic signaling is required for M2 function (e.g., B cell survival and expansion) *in vitro* but plays a less dominant role *in vivo*; and/or (iii) expression of IL-10 from an uninfected cell type (s) (e.g., macrophages or T cells) largely substitutes for the critical function of Y120.

In contrast, Y129 phosphorylation is possibly tightly regulated and active only upon antigen-dependent signaling and therefore plays a vital role in functions of M2 *in vivo* in the context of infection. If the latter is true, the discrepancy with the requirement of Y120 *in vitro* may be due to the absence of potential overlapping effector signaling molecules required by both the tyrosines in the *in vitro* settings, but substituted for or compensated by other factors in the *in vivo* setting. Indeed, *Pires de Miranda* have shown that in a latently infected B cell line, S11, Y129 is required for the interaction of M2 with PLCγ2 [24], an activator of calcium signaling required for activation of the NFAT pathway. Consistent with this, we have shown that the NFAT pathway is partially required for induction of IRF4, a key player in plasma cell differentiation [16]. It is therefore worthwhile to speculate that the phosphorylation status of M2 strongly dictates the outcome of M2-mediated functions.

Additionally, we also made mutations of Y120 and Y129 to aspartic acids in an effort to mimic constitutive phosphorylation. However, in the primary B cell assays, we found similar results as those observed with the Y→F mutations (data not shown). This might mean that either constitutive phosphorylation is deleterious to the function of the protein, rendering it unstable or that the mutations fail to mimic phosphorylation effectively. We did not pursue the Y→D mutations further since the focus of the study was aimed at understanding the requirement of Y120 and Y129 in M2-mediated function (s), rather than the effect of phosphorylation of M2 itself. It is also formally possible that the disconnect we observe with Y120 *in vitro* vs *in vivo* is due to the fact that most of the *in vitro* studies are done in B cell lines which are transformed, or in primary B cells that are stimulated with LPS. Under these conditions the status of the signal transduction pathways is likely dysregulated compared to B cell infection *in vivo*. This highlights the importance of extending tissue culture based findings to *in vivo* infection models.

Exploitation of the IL-10 pathway is a shared strategy among various virus families (reviewed in [34]). Several viruses are known to encode viral IL-10 homologues or induce cellular IL-10 as a means to simultaneously evade host immune response and enhance survival of infected cells. In particular, the human gammaherpesvirus EBV encodes a viral homolog, vIL-10 which is highly homologous (92.3% sequence identity) to cellular IL-10 and iscapable of enhancing transformation of human B cells [35]–[37]. In addition, the latent membrane protein-1 (LMP-1) of EBV induces cellular IL-10 via activation of the PI3K, Akt, NFkB pathway as well as activation of STAT3 [38]–[40]. Furthermore, serum IL-10 levels are increased in renal transplant patients undergoing EBV reactivation [41]. Although the role of IL-10 in KSHV pathogenesis remains unclear, KSHV encodes a viral IL-6 homolog which possesses overlapping functions with cellular IL-10 and IL-6. In macrophages and monocytes, KSHV microRNAs can increase cellular IL-10 and IL-6 secretion. Consistent with M2 phosphorylation of STAT3 (Figure 2), KSHV infection results in STAT3 activation in endothelial cells and dendritic cells (DCs) [42], [43]. In DCs, phosphorylation of STAT3 was also accompanied by an increase in the levels of IL-10, IL-6 and IL-23 [42]. Human betaherpesvirus CMV also encodes an IL-10 homolog, cmvIL10 [44] known to possess immunosuppressive roles [45]. cmvIL-10 also results in activation of STAT3 similar to that of EBV vIL10 and cellular IL-10, as well as M2-mediated IL-10 as described in Figure 2. Thus, IL-10 likely plays a dual role - an immunostimulatory role, as well as act as an immunosuppressive role depending on the cell type that it acts on. Taken together, the results from our study describe the residues on M2 that are required for IL-10 secretion and plasma cell differentiation and reactivation, thus aiding in better understanding the molecular basis of M2 mediated functions.

## Materials and Methods

### Ethics statement

This study was carried out in strict accordance with the recommendations in the Guide for the Care and Use of Laboratory Animals of the National Institutes of Health. The protocol was approved by the Emory University Institutional Animal Care and Use Committee and in accordance with established guidelines and policies at Emory University School of Medicine (Protocol Number: YER-2002245-031416GN).

### Mice and infections

Female C57BL6/J mice aged 6–8 weeks were purchased from Jackson labs (Bar Harbor, ME) and infections were done at 9–12 weeks of age. Mice were housed and maintained at the Whitehead vivarium according to Emory University and IACUC (International Animal Care and Use Committee) guidelines. Mice were anesthetized using isoflourane before infecting with 1000 PFU of the respective viruses via either intranasal or intraperitoneal infection. Splenocytes were harvested from mice that were sacrificed at the indicated time points by CO_2_ inhalation per AVMA guidelines. For flow cytometry analyses, individual mice were analyzed and for the latency and reactivation analyses, splenocytes from 3–5 mice per group were pooled.

### Plasmids

The Y120F and Y129F mutations were created by overlapping PCR using the following primers: Y120F–(5′GAAGAAAACATCTTCGAAACTGCTAAC 3′) and (5′GT TAGCAGTTTCGAAGATGTTTTCTTC 3′); Y129F–(5′ AGTGAACCAGTCTTCATCCAG CCAATC 3′) and (5′GATTGGCTGGATGAAGACTGGTTCACT 3′). The overlapping PCR products were then cloned into pCR-BLUNT (Invitrogen), sequenced and positive clones were then cloned into pMSCV-IRES-Thy1.1 vector as described in [17], using Bgl II restriction sites. The expression of M2/Y120F and M2/Y129F in pMSCV-IRES-Thy1.1 was verified by transfection of 293 T cells. Briefly, 60–80% confluent monolayers in 6 well plates were transfected with 5 ug of the plasmid using Mirus TransIT293 transfection reagent. Cell lysates were harvested at 48 hours and western blotting was performed as described in [16].

### Construction of mutant viruses

M2stop.HY and M2MR.HY viruses are described previously [16]. The Y120F.HY and Y129F.HY viruses were created in a similar manner. Briefly, the MHV68-H2bYFP BAC described in [29] was used as a backbone to create a M2/galK intermediate BAC where the M2 ORF was replaced with a galK cassette. The M2/galK intermediate was made by amplifying the galK gene from pGalK using the following primers – gK-M2-FP (5′-tggagggggtttcaacaggcactagtctgatgaggtttcgttttcaggtaTCAGC ACTGTCCTGCTCCTT-3′) and gK-M2-RP (5′-tccaggcgtgtttaaagaaaaagttatgttctgcgttagcaccttcactgCCTGTTGACAATTAATCATCGGCA-3′). These primers contain 50 bp homology arms that flank the M2 ORF. The resulting PCR product was transformed into the SW102/H2bYFP cells and screened by positive selection on minimal media containing galactose as the carbon source. Following confirmation of the M2/galK intermediate by restriction digest, the galK region was swapped out and replaced with either M2 or M2stop containing PCR products amplified from pMIT-Y120F or pMIT-Y129F plasmids using the same homology arms used for generating the M2/galK intermediate: M2-FP (5′-tggagggggtttcaa caggcactagtctgatgaggtttcgttttcaggtaATGGCCCCAACACCCCCACA-3′) and M2-RP (5′- tccaggcgtgtttaaagaaaaagttatgttctgcgttagcaccttcactgTTATATATAGCGATAGGTATCCTCCTCG-3′). After confirming the presence of required mutation by sequencing, the PCR products were electroporated into the M2/galK intermediate and recombinants were selected on minimal plates containing glycerol and 2-deoxy-D-galactose. Potential colonies were screened by colony PCR and confirmed by sequencing using primers spanning M2 ORF. RFLP analyses was also performed with SpeI and HindIII restriction endonucleases prior to final confirmation by Southern blotting using probes specific for the M2 ORF and its surrounding region.

### Flow cytometry

Flow cytometry on primary murine B cells were performed as described previously [17]. Briefly, cells were resuspended in about 100 uL of FACS buffer (PBS with 2% FCS+1 mM EDTA). Fc block was done prior to staining with fluorophores to block FcγIII/II receptors by adding Rat monoclonal anti CD16/32 (eBioscience) for 10 minutes. The cells were washed once with FACS buffer prior to staining with an antibody cocktail. Antibodies used for surface staining of primary B cells include Thy1.1-APC (eBioscience) and CD19-FITC. For staining splenocytes from infected mice, a similar protocol was used. Antibodies in the cocktail for infected cells include GL7-Ax660/APC (eBioscience), CD95-PECy7, CD138-PE, CD3, CD4, CD8-PerCP (BD Pharmingen), B220-Pacific Blue (Southern Biotech) described in [29]. CD3, CD4 and CD8 were used in the same fluorophore to eliminate T-cells. This mix is referred to in the text as dump gate.

### B cell isolation

Murine primary B cells were isolated by immunomagnetic negative selection using the EasySep Mouse B Cell Enrichment Kit (Stem Cell Technologies) as per manufacturer’s instructions. The purity of B cell isolation was routinely analyzed and found to be ≥95% as determined by staining for CD19 by flow cytometry. The cells were plated at 1×10^6^ cells/mL of a 24 well plate overnight with cRPMI containing 20–25 µg/mL LPS (Sigma) prior to retroviral transduction.

### Retroviral transduction

Retroviruses were prepared as described in [17]. Briefly, BOSC23 (ATCC) producer cells were plated at a density of 1.5×10^6^ cells per 60 mM or 3×10^6^ cells per 100 mM collagen coated dish (BD biosciences) overnight. After 18–24 hours of plating, the cells were transfected with 5 µg (for 60 mM) or 10 ug (for 100 mM dish) of either pMIT-M2, pMIT-M2stop, pMIT-Y120F or pMIT-Y129F plasmids. Supernatants were harvested at 72 hours and used immediately or frozen at −80°C until ready for use. On the day of transduction, the retroviruses were centrifuged at 2000 rpm for 10 minutes to remove any cellular debris and supplemented with 5 µg/mL of polybrene. 750 µL of the media was removed from the B cells and replaced with 1 mL of the retrovirus containing polybrene. The cells were spin infected for 2500 rpm for one hour at 30°C. Post-transduction, 750 µL of the supernatant was removed from each well and 1 mL of fresh media was added back. Triplicate wells per condition were analyzed by flow cytometry at days 2–5 post-transduction.

### Limiting Dilution analysis for latency and ex-vivo reactivation

Determination of frequency of latently infected splenocytes was done by performing a limiting dilution, single-copy sensitive nested PCR assay, as previously described [5], [46]. Briefly, frozen splenocytes were thawed, counted and washed in isotonic buffer prior to plating in serial three-fold dilutions onto a background of 10^4^ uninfected 3T12 cells in 96-well plates. Following a proteinase K mediated digestion step to lyse the cells, the samples were subject to two rounds of nested PCR. Twelve PCRs were performed for each dilution for a total of six dilutions starting with 10^4^ cells. Each PCR plate contained control reactions that contained 0, 0.1, 1, or 10 copies of plasmid DNA in a background of uninfected cells. Products were analyzed on a 2% agarose gel. Determination ofthe frequency of splenocytes capable of reactivating virus from latency was done by using a limiting dilution ex-vivo reactivation assay, as previously described [5], [46]. Briefly, single cell suspensions of splenocytes from infected mice were plated in a two-fold serial dilution fashion (starting with 10^5^ splenocytes per well) on to MEF monolayers in 96-well tissue culture plates. Twenty-four wells were plated per dilution and 12 dilutions were plated per sample. Wells were scored microscopically for cytopathic effect (CPE) at 14–21 days post-explant. Preformed infectious virus was detected by plating parallel samples of mechanically disrupted cells onto MEF monolayers alongside intact cells. We did not observe a significant amount of preformed infectious virus in any of the assays described (data not shown).

## Supporting Information

Figure S1 **Expression of M2 mutants.** Expression of Y120F and Y129F cloned into the pMSCV-IRES-Thy1.1 (pMIT) vector was tested by western blotting. Mutant retroviruses were prepared as described in materials and methods and transfected into 293T cells. Cell lysates were harvested at 48 h post transfection and western blotting was performed using chicken anti-M2 antibody described in [17].
(TIF)

Figure S2 **Southern blot analyses of mutant viruses.** M2MR.HY, M2stop.HY, Y120F.HY and Y129F.HY viruses were made as described in materials and methods. (A) Purified BAC preparations were digested with the restriction endonucleases SpeI or HindIII. Restriction fragments are shown. (B) The blot in (A) was subject to southern blotting using probes spanning the region encompassing M2.
(TIF)
