# Supplementary figures and images for: Tyrosine 129 of the Murine Gammaherpesvirus M2 Protein Is Critical for M2 Function In Vivo
Source: PLoS One. 2014 Aug 14;9(8):e105197. doi: 10.1371/journal.pone.0105197 (PMC4133380; doi:10.1371/journal.pone.0105197)

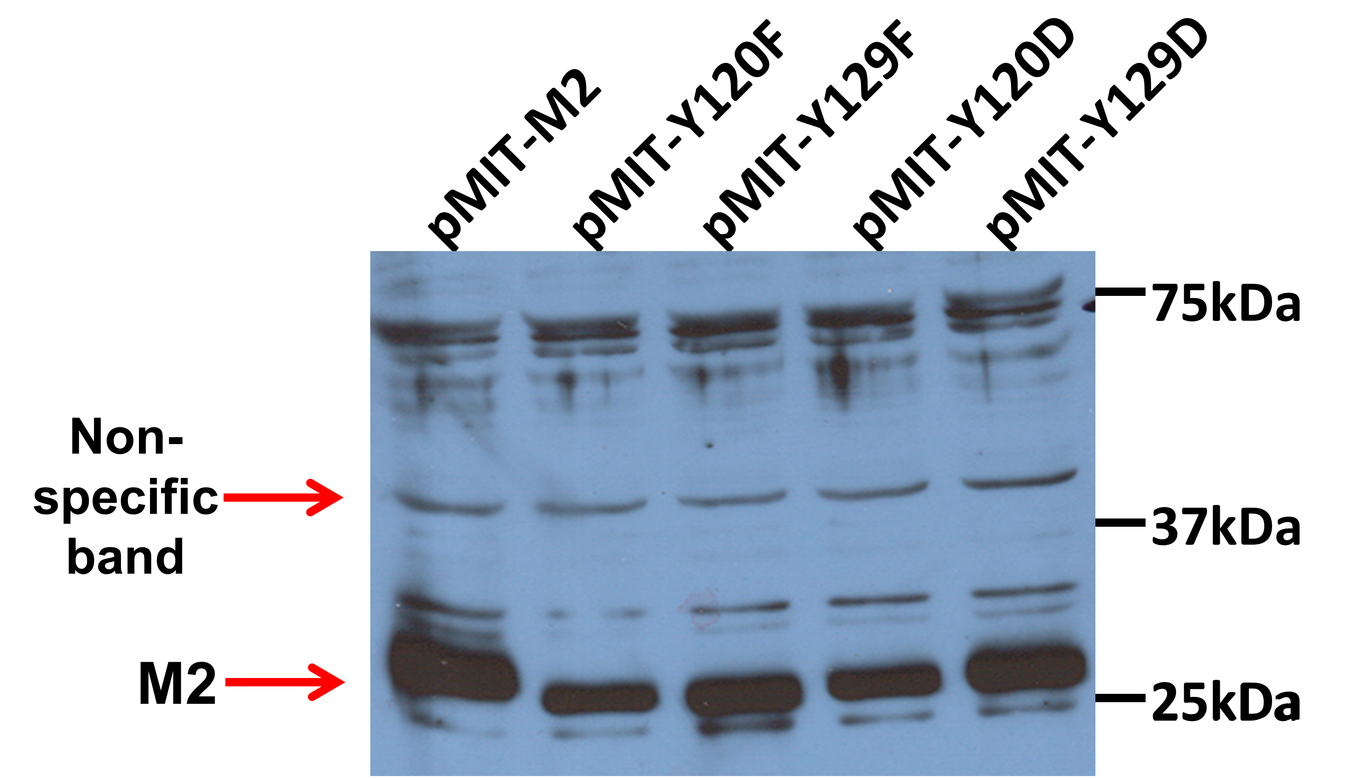

Supplement: Figure S1 — Expression of M2 mutants. Expression of Y120F and Y129F cloned into the pMSCV-IRES-Thy1.1 (pMIT) vector was tested by western blotting. Mutant retroviruses were prepared as described in materials and methods and transfected into 293T cells. Cell lysates were harvested at 48 h post transfection and western blotting was performed using chicken anti-M2 antibody described in [17]. (TIF) [file pone.0105197.s001.tif]

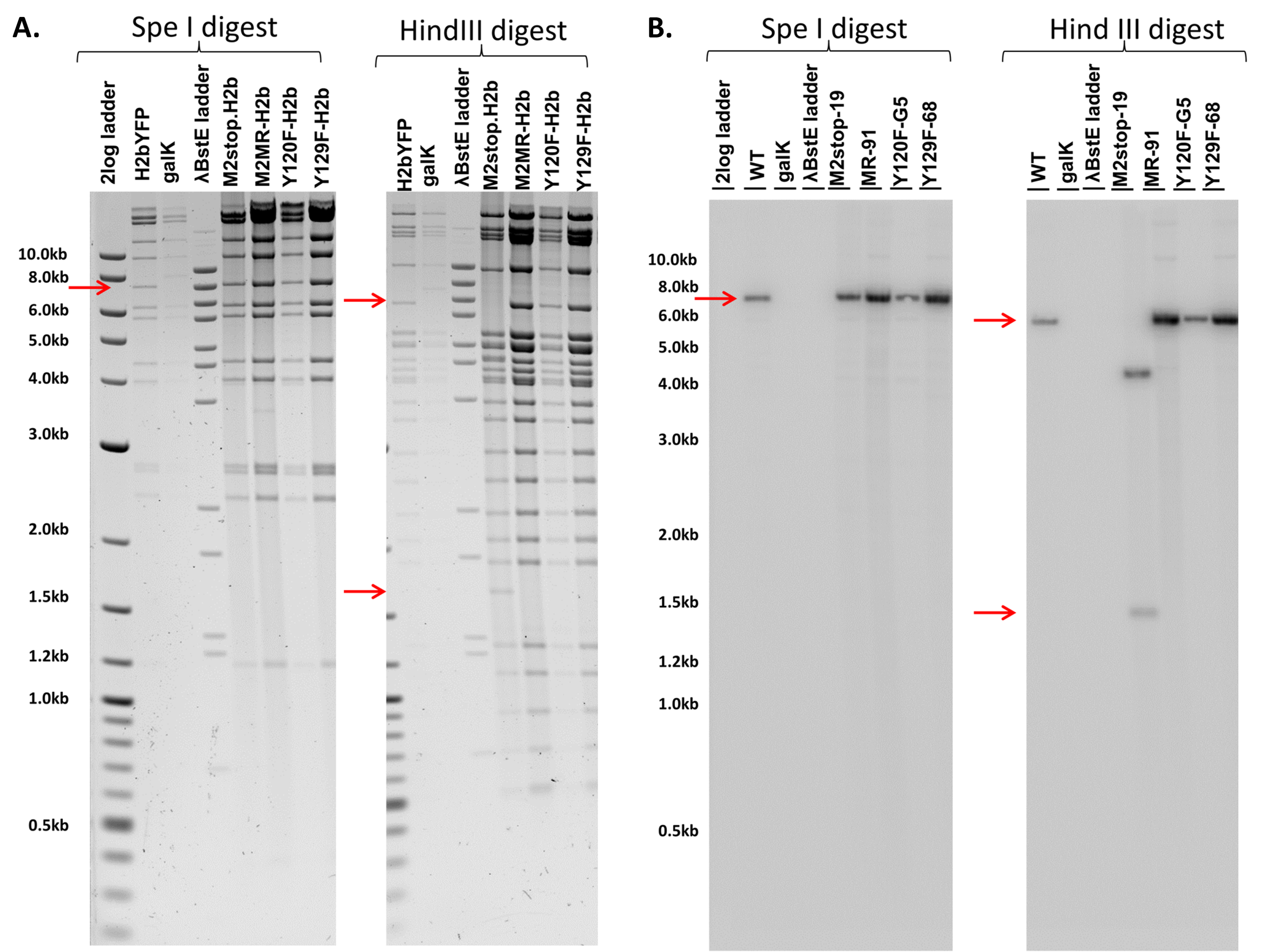

Supplement: Figure S2 — Southern blot analyses of mutant viruses. M2MR.HY, M2stop.HY, Y120F.HY and Y129F.HY viruses were made as described in materials and methods. (A) Purified BAC preparations were digested with the restriction endonucleases SpeI or HindIII. Restriction fragments are shown. (B) The blot in (A) was subject to southern blotting using probes spanning the region encompassing M2. (TIF) [file pone.0105197.s002.tif]
